# Effectiveness of Aquatic Therapy vs Land-based Therapy for Balance and Pain in Women with Fibromyalgia: a study protocol for a randomised controlled trial

**DOI:** 10.1186/s12891-016-1364-5

**Published:** 2017-01-19

**Authors:** Sabela Rivas Neira, Amélia Pasqual Marques, Irene Pegito Pérez, Ramón Fernández Cervantes, Jamile Vivas Costa

**Affiliations:** 1 Psychosocial Intervention and Functional Reabilitation Research Group. Physiotherapy Department, Faculty of Physiotherapy, University of A Coruña, Campus de Oza, A Coruña, 15006 Spain; 2 Department of Physical Therapy, Speech and Occupational Therapy, Faculty of Medicine, University of São Paulo, Rua Cipotânea, 51 – Cidade Universitária, São Paulo, 05360-160 Brazil

**Keywords:** Fibromyalgia, Physiotherapy, Pain, Postural Balance, Aquatic Therapy, Exercise

## Abstract

**Background:**

Fibromyalgia is a disease with an increasing incidence. It impairs the quality of life of patients and decreases their functional capacity. Aquatic therapy has already been used for managing the symptoms of this syndrome. However, aquatic therapy has only recently been introduced as a treatment modality for improving proprioception in fibromyalgia. The main objective of this study is to determine the effectiveness of two physiotherapy protocols, one in and one out of water, for improving balance and decreasing pain in women with fibromyalgia.

**Methods/Design:**

The study protocol will be a single-blind randomised controlled trial. Forty women diagnosed with fibromyalgia will be randomly assigned into 2 groups: Aquatic Therapy (*n* = 20) or Land-based Therapy (*n* = 20). Both interventions include 60-min therapy sessions, structured into 4 sections: Warm-up, Proprioceptive Exercises, Stretching and Relaxation. These sessions will be carried out 3 times a week for 3 months. Primary outcomes are balance (static and dynamic) and pain (intensity and threshold). Secondary outcomes include functional balance, quality of life, quality of sleep, fatigue, self-confidence in balance and physical ability. Outcome measures will be evaluated at baseline, at the end of the 3-month intervention period, and 6-weeks post-treatment. Statistical analysis will be carried out using the SPSS 21.0 program for Windows and a significance level of *p* ≤ 0.05 will be used for all tests.

**Discussion:**

This study protocol details two physiotherapy interventions in women with fibromyalgia to improve balance and decrease pain: aquatic therapy and land-based therapy. In current literature there is a lack of methodological rigour and a limited number of studies that describe physiotherapy protocols to manage fibromyalgia symptoms. High-quality scientific works are required to highlight physiotherapy as one of the most recommended treatment options for this syndrome.

**Trial registration:**

Date of publication in ClinicalTrials.gov: 18/02/2016. ClinicalTrials.gov Identifier: NCT02695875.

## Background

Fibromyalgia (FM) is a chronic disorder characterized by widespread pain in combination with tenderness in at least 11 of 18 established tender points [1]. In 2010, following the release of the new diagnostic criteria of the American College of Rheumatology (ACR) [2], a new definition for FM was adopted. In addition to pain, it includes other important symptoms such as fatigue, restless sleep, cognitive problems and a variety of somatic symptoms.

The mean prevalence rate of FM in Europe is 2.5% [3], and the prevalence in Spain in those greater than 20 years of age is 2.4% [4]. Worldwide, the incidence of FM is increasing at an exponential rate, demonstrating that FM is a common clinical condition that will require increasing specialised attention. FM causes disability, which is associated with high indirect costs derived from absenteeism and the use of health care resources. This is not only a major health problem, but also a significant socioeconomic problem [4]. There are many available treatments to manage FM, however, only a few are supported by scientific evidence. The latest report of the European League Against Rheumatism (EULAR) [5] agrees with current clinical practice guidelines (CPG) which state that aerobic exercise is the only treatment option for the management of FM based on strong scientific evidence. CPG from Germany and Israel consider aquatic therapy as the best aerobic exercise program for this type of patient [6] however, for the EULAR, the effectiveness of exercise does not depend on the environment where it is performed (in or out of water).

Both land-based and aquatic therapy programs have shown benefit in the treatment of FM. Land-based treatment includes different types of exercises that train aerobic ability, strength or flexibility [7] in a specific or general manner. Improvements in pain intensity, physical function and quality of life have been noticed. However, there are few studies with an appropriate methodological design that describe the intervention protocol and include physiotherapy as a treatment method. The properties of water make the aquatic environment one of the best places to carry out an exercise program: It reduces the impact on joints, improves microcirculation, facilitates relaxation, decreases the number of contractures and improves muscle tone due to its natural resistance [8]. Nevertheless, despite aquatic therapy showing benefits for the management of some FM symptoms, results should be analysed with caution because of the low methodological quality of studies [9].

Pain and quality of life are the most studied outcomes in FM. This protocol will focus on balance. Recently, it has been shown that patients with FM have problems with motor control [10] and postural balance [11]. Numerous observational studies have shown that there is a real balance disorder in patients with FM [12–14]. The results revealed patients’ difficulty to move quickly or to change center of body mass, while maintaining postural stability. Patients also perceive their own impaired balance, with 45% self identifying this problem as one of the ten most debilitating symptoms of FM [15]. Lack of self-confidence in balance seems to have a direct relationship with muscular strengh and an inverse relationship with pain, disease severity and frequency of falls [16].

Nowadays, health professionals agree that non-pharmacological therapy should be the first choice of treatment for FM, leaving the pharmacological therapy as a second-line intervention [5]. This fact reveals the importance of researching and developing new and effective forms of treatment that constitute reliable and safe alternatives for patients with FM.

The main objective of this study is to determine the effectiveness of two physiotherapy protocols for improving balance and decreasing pain in women with FM.

## Methods

### Study design

The study will be a single-blind randomised controlled trial.

### Enrollment and eligibility criteria

The sample will consist of 40 women, members of the “Fibromyalgia, Chronic Fatigue Syndrome and Multiple Chemical Sensitiviy Association” (ACOFIFA), in A Coruña (Spain).

### Inclusion criteria

- Female.
- Age range between 35 and 64 years [17].
- FM diagnosis according to the ACR criteria: 1990 [1] and 2010 [2].
- Mark ≥ 4 on “Visual Analogue Scale” (VAS) for pain.
- Mark ≥ 5 on EVA for balance, included in the “Revised Fibromyalgia Impact Questionnaire” (FIQR).

### Exclusion criteria

- Medical history of severe trauma.
- Neurological diseases.
- Frequent migraines.
- Diabetes.
- Severe psychiatric diseases.
- Peripheral nerve entrapment.
- Inflammatory rheumatic diseases.
- Pregnancy.
- People who suffered traumatic injuries in the past 6 months.
- Chlorine allergy.
- Anxiety conditions related to water.
- Infectious diseases.
- Severe cardiovascular disease.
- Heat intolerance.
- Patients who do exercise regularly.
- Significant changes in pharmacological treatment during the study period.

### Procedures

The assessments will take place at the Faculty of Physiotherapy of the University of A Coruña (Spain). Three assessment blocks will be established to carry out the measurements. A group of blinded trained assessors will be in charge of each block. In the first block, sociodemographic data (years since FM diagnosis, marital status, employment status, education level, smoking, number of falls in the last 6 months and medication) and anthropometric data (age, weight, height and body-mass index) will be registered. Pain intensity, fatigue, sleep quality, quality of life and self-confidence in balance will also be assessed. The second block will focus on measuring the pain threshold for the 18 tender points and functional independence in performing activities of daily life. Finally, the third block will assess physical ability and static/dynamic balance. Patients will be evaluated at three different moments: At baseline, immediately after the end of treatment and at 6-weeks follow-up.

### Randomisation

Once the patients have read and signed the informed consent, those who have met the inclusion criteria, will be randomly assigned to one of the two intervention groups:

- Active Control Group: Land-based exercise program (CG *n* = 20).
- Experimental Group: Water exercise program (EG *n* = 20).

**Fig. 1:**
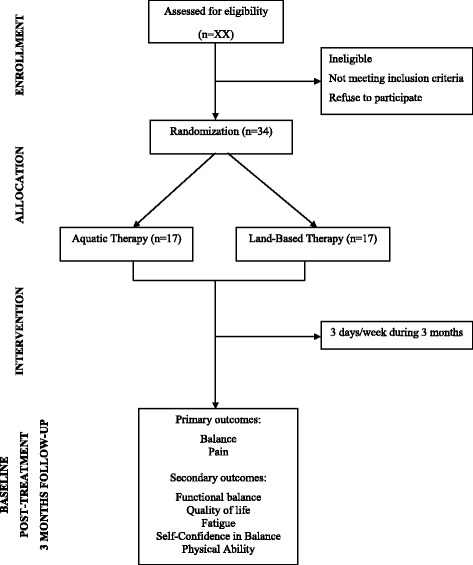
Flow diagram of the study

The randomisation will be carried out in a 1:1 manner via a computer-based scheme. The allocation will be concealed using sealed and opaque envelopes, numbered consecutively [18]. An independent researcher who will not participate in other study procedures will perform the randomisation process. The flow diagram of the study is summarised in Fig. 1.

### Outcome measures

All assessment instruments will be used in their validated Spanish versions, except for the Berg scale which is not validated in Spanish and will have to be applied in a translated version.

### Primary outcomes

#### Balance

Static balance will be assessed with the Romberg’s test [19] and dynamic balance with both the Timed Up & Go (TUG) test [20] and a gait test.

**Fig. 2:**
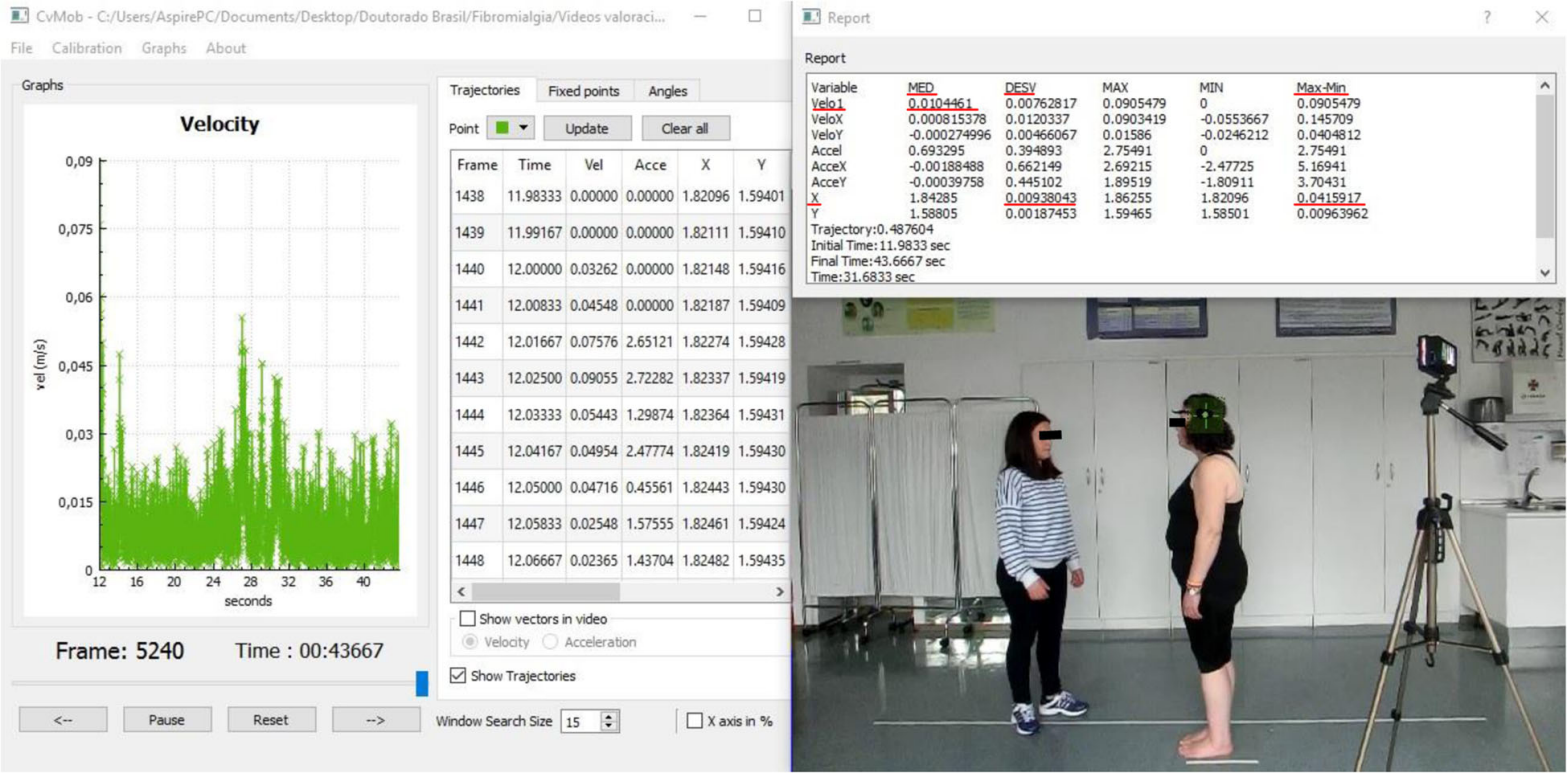
Analysis and data extraction for the oscillation around the AP axis in Romberg’s test

In order to quantify and increase the objetivity of balance assessment, all tests will be filmed. Mechanical parameters of the movement will subsequently be analysed with the Computer Vision Mobility (CvMob) software [21]; the analysis is shown in Fig. 2. Patients will be instructed to attend the study wearing a form fitting top and shorts, or swim suits [22] and to take off their shoes during all tests. The CvMob is an open source tool for movement analysis, created with the OpenCv and Qt libraries [23]. The software uses computing vision techniques, pattern recognition and optical flow to make object tracking possible, generating data of trajectory, speed, acceleration, and angular movement [23]. The equipment consists of a digital camera and CvMob program. The camera, a “Casio Exilim EX-ZR1000” model, with a resolution of 16.1 megapixels and 120 frames/s, will be used to record videos. The camera will be attached to a tripod and will be positioned at a distance of 2.27 m from the patient during the Romberg’s test and at 3.15 m for the gait test. For a proper analysis, the CvMob should always be calibrated at the begginning of each video. Calibration consists of providing a reference measurement to the software, which will be used to do all of the calculations. For the Romberg’s test, the instrument used to calibrate the system consists of a brown cardboard marked with two yellow points placed at a 20 cm interval distance. A mark painted on the floor of 21.5 cm in length will be used for the gait test. A series of markers will be placed on certain bony landmarks to facilitate registration of different motion parameters and further analysis. For static balance, the total speed, mean, standard deviation and amplitude of oscillation around the medio-lateral (ML) and anterior-posterior (AP) axes will be studied. The results that CvMob provides for these parameters are equal to those given by a conventional force platform. The gait speed and the length/height of step will be studied for dynamic balance. All of these parameters are explained in Table 1.

**Table 1:** Outcomes analysed with the CvMob software

Balance	Evaluation tool	Outcomes studied with CvMob	CvMob codes for the outcomes	Definitions of CvMob outcomes	Marker locations
Static	Romberg’s test	Oscillation speed	“Velo1 MED”^a^	Mean of the total speed oscillation (X-axis + Y-axis)	To analyze ML oscillation, a marker will be placed on the occipital bone, at the midpoint of the imaginary line passing between the tips of each ear. For AP oscillation, the marker will be located at the pterion, which is the junction between the parietal bones, frontal, sphenoid greater wing and squamous portion of the temporal bone.
		“X” mean	“X” MED”^a^	Representation of subject position in the video frame, on the X-axis.	
		Standard deviation	“X DESV”^a^	Representation of balance stability of the subject. Higher values indicate a greater imbalance regarding the patient’s centre. This index will depend on the “X” MED value.	
		Oscillation amplitude	“X max-min”^a^	Maximum amplitude of oscillation on the X-axis	
Dynamic	Gait test	Step length	“X max-min”^a^	Representation of step length.	Markers will be placed on the tip of internal and external malleolus. Markers are made of white foam rubber half-sphere stuck onto black tape, which allow for adherence to the patients’ skin.
		Step height	“Y max-min”^a^	Representation of step height.	
		Gait speed	“VeloX” MED^a^	Representation of total gait speed.	Markers will be located at the pterion. They will also be used for static balance: Yellow circular stickers with 20 mm diameter.

^a^This information is extracted from the software. The units of measures are in meters/s for the speed and in meters for the other outcomes

A recent study of validity and reliability [22] has shown that CvMob is a reliable tool for two-dimensional analysis of human gait. The results have revealed a strong correlation between CvMob and “Vicon Motion System” [24], a three-dimensional capture motion system with a high technological precision for movement analysis. In addition, a strong correlation has also been observed in both inter and intra-rater analysis. This demonstrates that CvMob results are reproducible by different researchers and by the same person, at different times [22].

#### Static Balance

Romberg’s test:

This test assesses the integrity of proprioception. Central postural control depends on three systems: Visual, vestibular and proprioceptive [25]. If the patient has a loss of proprioception, balance is maintained through activation of the visual and vestibular systems. However, if the patient is also deprived of eyesight, any proprioceptive disorder compensated by this system, will be detected and balance will be lost.

In order to increase test sensitivity, the Romberg’s test will also be performed with feet in the tandem position [19]. Therefore, 4 tests will be carried out, with a single attempt for each one and with a 10-s pause between each test:

- Test 1: Feet together, arms along the body and eyes open. Hold this position for 30 s.
- Test 2: Feet together, arms along the body and eyes closed. Hold this position for 30 s.
- Test 3: Feet in tandem position (the heel of the dominant must be placed inmediately in front of the non-dominant foot), arms crossed over the chest and eyes open. Hold this position for 30 s.
- Test 4: Feet in tandem position, arms crossed over the chest and eyes closed. Hold this position for 30 s.

The test is positive when the oscillation significantly worsens with the eyes closed [19, 26].

#### Dynamic balance

Timed Up & Go Test:

This test is a functional mobility test [20] whose purpose is to assess balance in the sitting position, transfers from a sitting position to a standing position and vice versa. It also evaluates stability during ambulation and direction changes while in gait without using compensatory strategies. The test consists of standing up from a chair with armrests and walking, at a normal speed, for 3 m, turning 180° and walking back to the chair. It will be practised once in order to insure that methodology is clear. At the time of assessment, only one single attempt will be registered. The test is measured in seconds and quantifies the time that the patient takes to complete the walk. A time of 10 s or less is considered normal and a time longer than 14 s is indicative of impaired balance and a high risk of falls [27].

Gait test:

The patient will have to walk, at a normal speed, for 8 m. The test will be practised once. As a limitation of optical range of the camera, only 3–4 gait cycles will be captured. Therefore, the 3 central meters of the walk should be used in the analysis of gait parameters.

### Pain

#### Pain intensity

It will be measured with the EVA, a 10 cm long line with the value 0 on the left indicating “no pain” and the value 10 on the right indicating the “worst imaginable pain” [28]. The distance along the line indicated by the patient will correlate with their average pain intensity in the last week. Scores between 0 cm and 3 cm are classified as “mild pain”; between 4 cm and 7 cm “moderate pain” and between 8 cm and 10 cm “severe pain”.

#### Pressure Pain threshold (PPT)

This is defined as the minimum pressure that triggers a painful response. An electronic algometer (Commander™ Algometer de JTECH Medical) will be used to measure the PPT on the 18 tender points, according to the ACR criteria [1]. The unit of pressure measurement will be kg/cm2, and the assessments will be done bilaterally, always beginning from the point located on the right. To avoid the risk of temporal summation [29], each tender point will be assessed only once. A 1 cm^2^ rubber tip will be used to centralise the pressure, the 18 tender points are:

- Occiput: Suboccipital muscle insertion.
- Supraspinatus muscle: Supraspinatus tendon, above medial scapular spine.
- Trapezius: Midpoint of the upper border.
- Greater trochanter: Posterior to the greater trochanter of the femur.
- Gluteus maximus: Upper outer quadrant of the buttocks in the anterior muscle fold.
- Lower cervical: Anterior C5-C7 intertransverse space.
- Second intercostal space: At the second costochondral junction.
- Lateral epicondyle: 2 cm distal to the lateral epicondyle.
- Medial knee: Medial fat pad of the knee, proximal to the joint line.

The procedure will be explained to the patients and demonstrated by performing a measurement on a non-included point. The rubber tip of the algometer will be placed perpendicularly to the skin and patients will have to say “stop” when the pressure begins to be painful.

### Secondary outcomes

#### Functional balance

This will be assessed with the “Berg Scale” [30], a 14-item scale that evaluates the static, dynamic and functional balance during the activities of daily living (ADL’s). Each item is scored from 0 to 4, where 0 means the inability to perform the task and 4 means the ability to complete the task without difficulty. The maximum score possible is 56 points and a score lower than 45 is related to risk of fall [31].

#### Quality of life

This will be assessed with the FIQR [32], a tool which tries to address the limitations of the Fibromyalgia Impact Questionnaire (FIQ) [33] while at the same time maintaining the basic properties of the FIQ. The FIQR is composed of 21 questions that make reference to the week prior to answering the questionnaire. Each question is based on an 11-point numeric rating scale of 0 to 10, with 10 being “worst”. The questionnaire is divided into three linked domains: Function, overall impact and symptoms. The “symptoms” domain contains four new questions relating to memory, tenderness, balance and environmental sensitivity (to loud noises, bright lights, odours and cold temperatures). The total FIQR score is the sum of the following 3 domain scores that can reach a maximum of 100 points: The “function” score (from 0 to 90) is divided by 3; the “overall impact” score (from 0 to 20) is not changed and the “symptoms” score (from 0 to 100) is divided by 2. Higher values indicate a poorer quality of life.

#### Quality of sleep

This will be evaluated with the Pittsburgh Sleep Qualitiy Index (PSQI) [34] a retrospective tool for measureing quality of sleep and sleep disorders. The PSQI is a 19-item questionnaire that refers to last month. It contains 7 sleep components: Subjective sleep quality, sleep latency, sleep duration, habitual sleep efficiency, sleep disturbances, use of sleeping medication and daytime dysfunction. The total PSQI is the sum of all component scores that can reach a maximum of 21 points. Higher values indicate a poorer sleep quality.

#### Fatigue

This will be evaluated by the Multidimensional Fatigue Inventory (MFI) [35], a 20-item assessment tool with five domains: General fatigue, physical fatigue, mental fatigue, reduced activity and reduced motivation. Each fatigue domain consists of four items and has a potential score ranging from 4 to 20, where higher MFI scores indicate a higher degree of fatigue.

#### Self-confidence in balance

This will be assessed with the Activities-specific Balance Confidence (ABC) scale [36], a 16-item questionnaire that measures the self-confidence in balance for performing ADL’s. Each item is based on a 0–100 scale where 0 is “no confidence” and 100 is “total confidence”. The total ABC score is calculated using the sum of all-items (range 0 to 1600) divided by 16. Scores >80% indicate a high level of physical functioning, 50–80% a moderate level, and scores <50% a low level of physical functioning. Scores <67% in older adults are predictive of future falls [37].

#### Physical ability

This will be measured with the 6-minute walk test [38], which determines the maximum distance that a person can walk in 6 minutes along a 20-m corridor. Heart rate (HR) and oxygen saturation will also be assessed with pulse oximetry. Dyspnea and lower limb fatigue will be measured with the modified Borg scale [39]. These parameters will be registered before the start of the test, immediately after and during recovery time (when the patient returns to baseline HR).

### Interventions

The interventions designed in this protocol consist of two similar physiotherapy protocols for people with FM. Both will include 60-min sessions that will be carried out 3 times a week for 3 months by a physiotherapist, in groups of 8–9 people maximum.

Both interventions will be based on: 15 min of warm-up, 25 min of proprioceptive exercises, 8 min of stretching and 12 min of relaxation.

For adequate training of balance and postural control, patients will be required to contract their local musculature (“core stability”) before starting any specific exercise. The transversus abdominis, pelvic floor muscles, internal oblique and multifidus form the local musculature. The most important aspect of achieving core stability will be co-activation of the first two muscles, for which patients will have to place their pelvis in a neutral position. Before starting the interventions, patients will receive anatomy and palpation classes to aid in identification of the involved musculature and how its contraction is perceived.

The protocols have been created by the main researcher based on available scientific evidence. The protocols were designed with the intention of being as similar as possible in order to attribute any statistically significant difference in outcomes between the two groups to the environment where the interventions were performed. Sessions will be pre-programmed with a progression in difficulty over the intervention period: Shorter pauses, higher exercise intensity, eyes closed, etc.

Patients will not be allowed to begin any other activity during the study period. They will have to report any problems, whether event-related or not, as well as any medication changes.

### Aquatic therapy

The twenty patients included in the EG will perform aquatic therapy in the Rialta Sports Complex, in A Coruña (Spain). The water temperature is 30 °C, with less than 1 °C of variation, and the environmental temperature is 27.5 °C, with less than 1 °C of variation. Sessions will be given in a swimming-pool of 20 × 6 m, with a 120-cm depth. The aquatic therapy protocol is described in Table 2.

**Table 2:** Description of aquatic therapy protocol

Exercise blocks	Exercise descriptions	Repetitions/Action/Pause
Warm-up (15 min)	1. Running in water: With water at waist level, patients will run along the bottom of the pool, changing trajectory. 2. Can-Can kicks: Submerged to chest depth, patients will kick the water with alterating legs. 3. Hydro-Jumps: With feet on the pool’s floor, patients will jump, bending their knees at the highest point in their jump. 4. Pedaling: With a pool noodle under the neck, patients will move their legs in the motion of pedaling a bicycle while moving along the pool. 5. Rocking Horse: With one foot before the other, patients will alternate jumps with the front and back leg. 6. Relay Race: 2 groups. The winner will be the group that returns the baton to the first participant in the shortest time.	1. 3 min uninterrupted activity. 2. 3/30 s/15 s. 3. 3/30 s/15 s. 4. 3 min uninterrupted activity. 5. 3/30 s/15 s. 6. 2/1 min/20 s.
Proprioceptive exercises (25 min)	1. Playing Catch: In a group, patients will be sitting on a pool noddle and will have to maintain balance while throwing and catching the ball.	1. 3 min uninterrupted activity.
	2. Balance over pool noodles: Patients will be sitting on a pool noodle with hips and knees bent 90° and will have to keep balance in 3 different positions: a. With arms submerged and 90° abduction; b. one arm out of water and the other under water; c. from the initial position, they will have to do a trunk extension with shoulder extension, hip extension, knees pointing to the pool’s floor and neck extension.	2. 2 (for each position)/1 min/20 s.
	3. Turbulence standing: Standing, with water at the level of the chest and arms along the body; patients will have to do quick and short flexion/extension movements with the ULs generating significant turbulence. A good activation of local musculature will be essential for avoiding imbalance.	3. 4/15 s/15 s.
	4. Exercises with kickboard: a. Patients will be sitting on a kickboard, with water at the height of neck. They will have to keep afloat with only the aid of pedaling and without moving along the pool. b. With one foot on the kickboard and the other on the pool’s floor, patients will have to lower the kickboard and place it 10 cm above the pool’s floor. They will have to maintain this position and move the kickboard forward, backward and sideways without allowing it to go to the water’s surface. The exercise will be done with both LLs, first placing the kickboard vertically and then horizontally.	4. a. 2/1 min/20 s.; b. 2/40 s/15 s.
	5. Double pool noodle: Patients will be standing with a pool noodle in each hand. They will have to submerge them in the water while raising knees to chest.	5. 3/50 s/20 s.
	6. The boat: 2 groups. Patients will have to submerge a large mat while maintaining a standing position with different supports: Double, single-leg and tandem.	6. 3 min uninterrupted activity.
Stretching (8 min)	Gastrocnemius, quadriceps, ischiotibial, adductors, quadratus lumborum, deltoid, triceps brachii, superior trapezius.	2 (right and left side)/30 seg/5 seg.
Relaxation (12 min)	An Ai-Chi sequence with music. 6 movements of the 19 that comprise the Ai-Chi done in the following order: “Folding”, “Soothing”, “Gathering”, “Freeing”, “Shifting” and “Accepting”.	12 min uninterrupted activity.

*ULs* upper limbs, *LLs* lower limbs

### Land-based therapy

The twenty patients included in the CG will perform the intervention in one of the laboratories at the Faculty of Physical Therapy. The land-based therapy protocol is described in Table 3.

**Table 3:** Description of land-based therapy protocol

Exercise blocks	Exercise descriptions	Repetitions/Action/Pause
Warm-up (15 min)	1. Vigorous Walking: Patients will have to walk forwards, backwards, snaking and changing direction energetically. 2. Standing exercises: a. On one leg, with an UL and its contralateral LL, patients will do a simultaneous abduction followed by addution. b. The shoulder flexion movement is combined with contralateral knee elevation. c. Patients will perform jumping jacks. 3. Ball Jumps: Patients will be sitting on a Bobath ball and will have to jump. 4. Pedaling: Patients will be positioned face up, with the hips and knees bent 90° and will have to do a pedaling motion with their legs, keeping the pelvis in a neutral position. 5. Relay Race: 2 groups. The winner will be the group that return the baton to the first participant in the shortest time.	1. 3 min of uninterrupted activity. 2. a. and b. 6 (3 for each diagonal)/15 s/5 s; c. 3/20 s/10 s. 3. 3/45 s/20 s. 4. 3/45 s/20 s. 5. 3/1 min/20 s.
Proprioceptive exercises (25 min)	1. Playing Catch: Patients will be placed in a circle and sitting on a Bobath ball. With single-foot support, they will have to maintain a good position while throwing and catching the ball.	1. 3 min of uninterrupted activity.
	2. The bridge: a. Patients will be positioned face up on a mat, with arms along the body and feet on a Bobath ball. They will have to raise their buttocks off the floor and hold this position (the bridge). b. Starting from the previous position, patients will do the bridge raising one of the LL supported on the ball together with the contralateral UL at the same time.	2. a. 2/1 min/20 s.; b. 4 (2 for each diagonal)/40 s/5 s.
	3. The knight: With one knee on a Dynair and the contralateral foot on a hedgehog (Erizo Senso® Balance), patients will have to maintain the position without losing balance.	3. 6 (3 for each side)/30 s/10 s.
	4. Standing balance: Standing on a Dynair, patients will have to maintain their balance while moving their center of gravity forward, backward and sideways. There can not be any contact with the floor through the Dynair. The exercise will also be performed on one leg.	4. 3/1 min/15 s.
	5. Superman: On all fours, with hands holding a roll (SISSEL® Pilates Roller), patients will have to perform and hold the superman position (simultaneous extension of an UL and contralateral LL). The exercise will also be performed dynamically: Hand touching the knee and then moving away, while keeping the pelvis in a neutral position.	5. 8 (4 for each diagonal: 2 dynamic and 2 keeping the position)/30 s/10 s.
	6. Exercises with the roll (SISSEL® Pilates Roller): a. Patients will be sitting on one of the roll’s ends and must move their trunk backward (reaching the stability limit), while keeping both feet fully supported on the floor. b. With the spine resting on the roll and hands and feet on the floor, patients will place their hips and knees in a flexion of 90°, without losing balance. This exercise will also be performed dynamically (raising and lowering the legs) and removing one of the hand supports.	6. a. 3/15 s/10 s.; b. 4 (2 dynamic y 2 keeping the position)/50 s/10 s.
Stretching (8 min)	Gastrocnemius, quadriceps, ischiotibial, adductors, quadratus lumborum, deltoid, triceps brachii, superior trapezius.	2 (right and left side)/30 s/5 s.
Relaxation (12 min)	Jacobson Progressive muscle relaxation, with classical music.	12 min of uninterrupted activity.

*UL* upper limb, *LL* lower limb, *Dynair* Balance Soft Disc

### Statistical issues

#### Sample size calculation

The sample size was calculated to find a difference of ± 2.5 points between intervention groups on the VAS pain intensity scale [40], with a standard deviation of 2.5 points [41].

In order to achieve a statistical power of 80% with a significance level of *p* ≤ 0.05 and assuming a 20% dropout rate, an estimated 20 participants are required in each of the intervention groups. This sample size allows for detecting differences of 2 ± 2 s in the TUG test, with a statistical power of 80% and a significance level of *p* ≤ 0.05, assuming a 20% dropout rate.

The sample size was defined for a bilateral hypothesis and was carried out by the ENE software.

### Statistical analysis

Analysis will be descriptive of all outcomes included in the study, expressing quantitative outcomes with their mean ± standard deviation and qualitative outcomes with their absolute value, percentage and 95% confidence intervals.

The association between qualitative outcomes will be studied using the Chi-square test. After checking normality with the Kolmogorov-Smirnov test, the Student *T* test or the Mann–Whitney *U* test will be used to perform mean comparison. The mean comparison between two or more categories will be studied with the ANOVA test or Kruskal-Wallis test, as appropriate.

The correlation between quantitative outcomes will be analysed with the Pearson or Spearman correlation coefficients, as appropriate.

The mean comparisons for related outcomes in two different moments will be studied with the Wilcoxon test. Friedman test will be used when comparing more than two moments. In addition, the clinical relevance of the intervention will be studied by calculating the relative risk, relative risk reduction, absolute risk reduction and the number needed to treat. All of these measures will be presented with their 95% confidence interval.

A multivariate analysis by multiple linear regression or logistic regression to adjust for the effectiveness of the intervention according to possible confounding factors and to determine what other outcomes might be associated with each result will be carried out. Only the outcomes that show a statistical significance p <0.20 in the bivariate analysis, will be included in the multivariate regression analysis. In addition, a stepwise backward modelling strategy will be carried out.

All analysis will be done by intention to treat [42], where the total value of randomisation is preserved and control of any counfounders’ effect is insured.

The significance level set for all the analysis will be *p* ≤0.05. The SPSS statistical software, version 21.0 (SPSS, Chicago, IL) will be used for all analysis.

## Discussion

The main objective of this randomised controlled trial is to determine the effectiveness of two physiotherapy protocols in improving balance and decreasing pain in women with FM, at the end of the intervention and at 6-weeks follow-up. With the study conclusion, we expect to test the following null hypothesis: “There is no difference in balance or pain for participants undergoing physiotherapy interventions on land or in water”. The balance disorder observed in FM is a sympton that has been discovered only recently. This is why there are very few publications regarding. Specifically, in the MEDLINE database, we were able to identify only 11 randomized controlled trials that included physical interventions and balance improvement was included in their objectives. Of these 11 clinical trials, only three included physiotherapy as a treatment method [43–45]. Given this, we expect the study conclusion to contribute to the fund of scientific knowledge, providing evidence that physiotherapy is a safe and effective tool in the management of FM symptoms, specifically balance disorders and pain.

## Abbreviations

ABC: Activities-specific balance confidence
ACOFIFA: Fibromyalgia, chronic fatigue syndrome and multiple chemical sensitiviy association
ACR: American College of rheumatology
ADL’s: Activities of daily living
AP: Anterior-posterior
CG: Control group
CPG: Clinical practice guidelines
EG: Experimental group
FIQ: Fibromyalgia impact questionnaire
FIQR: Revised fibromyalgia impact questionnaire
FM: Fibromyalgia
HR: Heart rate
MFI: Multidimensional fatigue inventory
ML: Medio-lateral
PSQI: Pittsburgh sleep qualitiy index
TUG: Timed up & go test
VAS: Visual analogue scale
